# The Effects of a 6-Month High Dose Omega-3 and Omega-6 Polyunsaturated Fatty Acids and Antioxidant Vitamins Supplementation on Cognitive Function and Functional Capacity in Older Adults with Mild Cognitive Impairment

**DOI:** 10.3390/nu12020325

**Published:** 2020-01-26

**Authors:** Pinelopi S. Stavrinou, Eleni Andreou, George Aphamis, Marios Pantzaris, Melina Ioannou, Ioannis S. Patrikios, Christoforos D. Giannaki

**Affiliations:** 1 Department of Life and Health Sciences, University of Nicosia, Nicosia 2417, Cyprus; stavrinou.p@unic.ac.cy (P.S.S.); andreou.el@unic.ac.cy (E.A.); aphamis.g@unic.ac.cy (G.A.); 2 University of Nicosia Research Foundation, Nicosia 2417, Cyprus; 3 The Cyprus Institute of Neurology and Genetics, Nicosia 2371, Cyprus; pantzari@cing.ac.cy; 4 Noesis Cognitive Center, Materia Group, Nicosia 2221, Cyprus; melina@noesis.eu.com; 5 School of Medicine, European University Cyprus, Nicosia 2404, Cyprus; i.patrikios@euc.ac.cy

**Keywords:** antioxidant vitamins, cognitive function, elderly, functional capacity, mild cognitive impairment, PUFAs, supplementation

## Abstract

The aim of the present study was to examine the effects of a high-dose omega-3 and omega-6 fatty acids supplementation, in combination with antioxidant vitamins, on cognitive function and functional capacity of older adults with mild cognitive impairment (MCI), over a 6-month period in a randomized, double-blind, placebo-controlled trial. Forty-six older adults with MCI (age: 78.8 ± 7.3 years) were randomized 1:1 to receive either a 20 mL dose of a formula containing a mixture of omega-3 (810 mg Eicosapentaenoic acid and 4140 mg Docosahexaenoic acid) and omega-6 fatty acids (1800 mg gamma-Linolenic acid and 3150 mg Linoleic acid) (1:1 *w*/*w*), with 0.6 mg vitamin A, vitamin E (22 mg) plus pure γ-tocopherol (760 mg), or 20 mL placebo containing olive oil. Participants completed assessments of cognitive function, functional capacity, body composition and various aspects of quality of life at baseline and following three and six months of supplementation. Thirty-six participants completed the study (eighteen from each group). A significant interaction between supplementation and time was found on cognitive function (Addenbrooke’s Cognitive Examination -Revised (ACE-R), Mini-Mental State Examination (MMSE) and Stroop Color and Word Test (STROOP) color test; *p* < 0.001, *p* = 0.011 and *p* = 0.037, respectively), functional capacity (6-min walk test and sit-to-stand-60; *p* = 0.028 and *p* = 0.032, respectively), fatigue (*p* < 0.001), physical health (*p* = 0.007), and daily sleepiness (*p* = 0.007)—showing a favorable improvement for the participants receiving the supplement. The results indicate that this nutritional modality could be promising for reducing cognitive and functional decline in the elderly with MCI.

## 1. Introduction

Mean life expectancy is projected to increase and both the proportion and absolute number of older people in populations around the world are increasing dramatically [1,2]. This extended life should grow in parallel with an increased disease-free lifespan (health span); therefore, policies that can support healthy ageing are necessary [3,4]. Increasing age is associated with declines in both cognitive and physical function [5]. Cognitive function and functional capacity are major determinants of quality of life in the elderly, and a reduction in these two factors may affect their ability to carry out activities of daily living. There is a close link between physical frailty and cognitive decline with respect to their major underlying mechanisms, such as chronic inflammation, oxidative stress and neuroendocrine dysfunction [5]. Therefore, strategies that can slow the age-related decline of these functions are needed to prevent severe cognitive impairment and functional inability, and to help older adults maintain adequate performance status and independence, thus approaching the goal of healthy ageing.

Polyunsaturated fatty acids (PUFAs) have attracted great attention for their health-enhancing effects as well as specific vitamins for their strong antioxidant abilities [6,7]. PUFAs such as omega-3 and omega-6 fatty acids are fatty acids with two or more double bonds in their carbon chain backbone. Omega-6 fatty acids include linoleic acid (LA), γ-linolenic acid (GLA) and arachidonic acid (AA). Omega-3 fatty acids include eicosapentaenoic acid (EPA) and docosahexaenoic acid (DHA). However, these fatty acids should be consumed from the diet or through supplementation since their synthesis is limited in humans [6,8].

Cell membrane fatty acid composition can be modified with dietary supplementation, but the process is age dependent (taking longer in adult vs. developing brains) and may be dependent on the quantity of the dietary/supplemented PUFAs [9]. Diets high in DHA /EPA and or LA/GLA can increase the proportion of these PUFAs in the membranes of inflammatory cells, and also reduce levels of AA, a stress-related biomarker and inflammatory process initiator (through pro-inflammatory eicosanoids production) [10,11].

Furthermore, vitamin E is an important antioxidant that can quench free radicals [12]. Specifically, vitamin E (as α-tocopherol isoform) can very efficiently detoxify hydroxyl, perhydroxyl and superoxide free radicals, whereas γ-tocopherol (different isoform of vitamin E) has been shown to be more efficient in trapping NO radicals [13,14]. Additionally, α-tocopherol is associated with non-antioxidant properties, like the modulation of cell signaling, the function of the immune system and the induction of apoptosis [15].

The potential benefits of PUFAs, especially of EPA and DHA, in preventing cognitive decline have been of clinical interest, due to their neuroprotective properties such as increase neuroplasticity of nerve membranes, promoting synaptogenesis, modulation of signal transduction pathways in neuronal cells and attenuating inflammation [6,8,16]. However, not all studies in the precedent literature are in agreement concerning the efficacy of PUFAs supplementation on cognitive function, with some meta-analyses reporting positive effects, and others finding no effects [17,18,19,20]. The reasons for these discrepancies between studies could possibly be due to the different amount of PUFA (both omega-3/omega-6) dose given, the weight ratio between the individual PUFA in the formulation used, the type and quality of the source of PUFA used (as fish oil and/or vegetable oil, or else) but also on the homogeneity of the population in the trial and the limitations of each of the trials and the various cognitive tests employed, as well as the diversity of participants regarding age and cognitive status.

In 2015, the World Health Organization (WHO) in its world report on ageing and health, defines healthy ageing as “the process of developing and maintaining the functional ability that enables well-being in older age” [2]. Moreover, it is well recognized that muscle weakness and poor functional ability are strong predictors of clinically relevant adverse outcomes in older adults including falls, dementia, nursing home admission and mortality [21,22,23,24]. In the last decade, there have been numerous studies demonstrating a positive impact of EPA and DHA supplementation on skeletal muscle strength and mass, even though this was not confirmed in other studies [25,26,27,28,29]. However, only few studies have examined the effect of omega-3 fatty acid supplementation on the physical performance of elderly people [27,28,29,30,31]. Nevertheless, based on these limited data of various doses and supplementation duration, there is conflicting evidence as to whether omega-3 alone or in combination with omega-6 supplementation could confer a beneficial effect on muscle function in older adults as a monotherapy and without an exercise component.

Therefore, given that there is a close relationship between cognitive and physical decline in the elderly, it is important to find interventions targeting both of these major concerns for older adults. The primary aim of the present study was to examine the effects of high-dose omega-3 and omega-6 fatty acids supplementation—in combination with antioxidant vitamins—on the cognitive function and functional capacity of older adults with mild cognitive impairment (MCI) over a 6-month period. The secondary aims included the investigation of the effects of the above intervention on quality of life and wellbeing related parameters such as sleep quality, daily sleepiness and fatigue.

## 2. Materials and Methods

### 2.1. Participants

**Figure 1:**
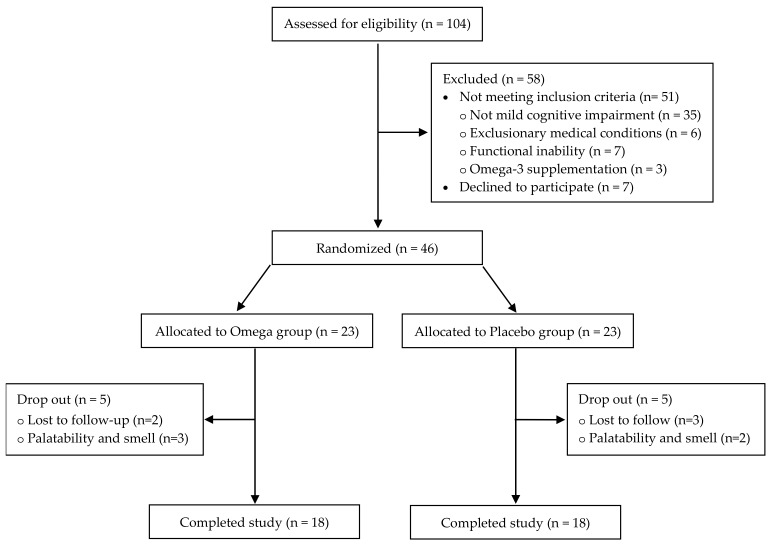
Flow of participants through the study.

The study was a 6-month randomized double-blind placebo-controlled study among older adults with MCI. Participants were recruited from long-term care facilities, medical centers, neurology clinics and community centers. One-hundred-and-four Caucasian older adults (≥ 65 years old) were assessed for eligibility. All participants gave written informed consent at the time of enrolment. Individuals with probable MCI, according to the criteria of Petersen, (memory complaint usually corroborated by an informant, objective memory impairment for age, essentially preserved general cognitive function, largely intact functional activities, not demented) were included in the current study [32]. Face-to-face interviews were conducted by qualified investigators using the Addenbrooke’s Cognitive Examination -Revised (ACE-R) test to screen cognitive impairment. In addition, the inclusion criteria were as follows: (1) an ACE-R score ≤ 88 [33,34]; (2) having no significant medical condition (e.g., cancer or cerebrovascular disease); (3) no previous use of omega-3 and/or omega-6 and/or vitamin A or E supplementation. All analyses were performed per protocol, for the entirety of the study on the entire study population. In total, forty-six older adults with MCI were found to be eligible to be enrolled in the study. Five participants of each group dropped-out (lost to follow-up or due to palatability and smell problem) and eighteen in each group completed the study (Figure 1). The study was approved by the National Bioethics Committee (ΕΕΒΚ/ΕΠ/2017/24) and was in accordance with the declaration of Helsinki.

### 2.2. Study Design

An independent researcher not involved in data collection or analysis performed the computer-generated randomization according to age and ACE-R score. The investigators and research personnel, nursing and medical personnel and participants were blinded to treatment allocation throughout the study. The supplementations had identical appearances and smells and were kept in dark bottles labelled with code numbers, blinded for both the participants and investigators.

Participants were randomized to receive either a daily dose of a 20 mL cocktail formula (Neuroaspis PLP10^®^) consisting of omega-3 ((EPA (810 mg)/DHA (4140 mg)), omega-6 ((GLA (1800 mg)/LA (3150 mg)) (1:1 *w*/*w*), vitamin A (0.6 mg), vitamin E (22 mg as α-tocopherol) plus pure γ-tocopherol (760 mg) and citrus-aroma or 20 mL of placebo (pure virgin olive oil) [35]. The intervention and placebo were isocaloric. The supplementations were taken orally once daily 30 min before dinner using a dosage-calibrated cup for 6 months.

All participants were asked not to change their current dietary and physical activity habits during the study period. Cognitive function, functional capacity, body composition, quality of life, sleep quality, daily sleepiness and fatigue assessments were held over 3 different testing days (within the same week) at baseline and following 3 and 6 months of supplementation. As indicated previously, all analyses were performed per protocol (on all time on study population).

### 2.3. Outcome Measures

#### 2.3.1. Cognitive Function

The ACE-R, Mini-Mental State Examination (MMSE), Trail Making Test (TMT), Stroop Color and Word Test (STROOP) and a symbol cancellation test were used to evaluate the cognitive function of the participants.

ACE-R test contains five subscores, each one representing a cognitive domain: attention/orientation (18 points), memory (26 points), fluency (14 points), language (26 points) and visuospatial (16 points), giving thus a maximum score of 100 points [34,36]. ACE-R incorporates the MMSE test (30 points). The sensitivity and specificity of this test for detecting MCI has been previously shown [37,38]. The test lasted 15-20 min for each participant.

The TMT provides information on visual search, scanning, speed of processing, mental flexibility and executive functions. The TMT consists of two parts. In Part A, the participants were required to draw lines sequentially connecting 25 encircled numbers distributed on a sheet of paper. In Part B, the participants were instructed to connect numbers and letters in alternating and ascending order (e.g., 1–A–2–B –3–C, etc.). The score on each part represents the amount of time required to complete the task [39,40].

The STROOP test was selected as a measure of inhibition, a key process of executive function [41]. The Stroop test consists of speeded trials of word reading, color naming, and color-word interference; each lasting 45 s. The first trial (word) assessed the number of color words that can be read aloud as quickly as possible (i.e., names of colors—red, green, and blue printed in black ink). In the second trial (color), the participants were asked to look at a series of color stimuli and the number of correctly named colors was assessed (i.e., a string of X’s printed in one of the three respective colors). In the third trial (color-word), the participants were asked to look at a series of color words, appeared in incongruent ink colors, and name the color of the ink instead of reading the word (e.g., the word ‘‘RED’’ printed in blue ink). The scoring of each trial relied on the number of correct responses. In addition, an interference score was calculated by subtracting the predicted color–word score from the obtained color–word score [41]. A lower score represents a greater difficulty in inhibiting interference.

In the symbol cancellation test, the participants were asked to identify 60 target stimuli that were embedded in a background of over 300 distractor stimuli, in 45 s. Each target omission or incorrectly identified distractor was marked as 1 error [42].

The TMT, STROOP and symbol cancellation tests were only executed at baseline and at 6 months following supplementation. A lower number of participants were included in the analysis of these tests due to various reasons (e.g., reduced visual function, unable to complete the test).

#### 2.3.2. Functional Capacity

Functional capacity was evaluated using three sit-to-stand (STS) tests (STS-5, STS-30 and STS-60), the timed–up-and-go (TUG) test, the 6-min walk test (6MWT), and the handgrip strength (HGS) test. The sit-to-stand tests measure lower body power, balance and endurance and require participants to rise from a chair with their arms across their chest and sit down again [23,43]. The STS-5 record the amount of time (in seconds) to complete five sit-to-stand cycles, while the STS-30 and STS-60 score is the total cycles (repetitions) achieved in 30 and 60 s, respectively. The TUG test is a functional mobility measure that mainly assesses gait and dynamic balance. The participant is timed while he rises from a chair, walks 3 m, turns, walks back, and sits down again [44]. The 6MWT examines physical performance and endurance of the subjects. It requires from the participant to cover as much ground as possible in six minutes and the total distance (meters) walked is measured [45]. HGS is a widely used method for measuring muscle strength [46]. Participants were asked to squeeze the digital dynamometer (T.K.K. 5401 Grip-D; Takey scientific instrument co., Ltd.) as hard and as tightly as possible for 3–5 s. Two measurements for each hand, alternating sides, were performed and the best of grip strength measurements was reported as the final result.

#### 2.3.3. Body Composition

Body mass was measured to the nearest 0.1 kg using a portable analogue scale (Seca model 755, Hamburg, Germany) and height was measured using a standing stadiometer (Seca model 720, Hamburg, Germany) with precision of 1 mm. Body mass index, fat mass index and lean mass index were calculated as body mass, fat mass and lean mass (kg) divided by height (m^2^), respectively. Trunk fat percentage and waist circumference were measured using a validated bioelectrical impedance device (Viscan abdominal fat analyzer AB140, Tanita Corporation, Tokyo, Japan). Total body fat percentage and total body water was measured with bioelectrical impedance analysis (Quadscan 4000, Bodystat Ltd., Douglas, UK). Lean body mass was estimated by subtracting body fat weight from total body weight. Hip circumference was measured to the nearest 0.1 cm using a flexible tape measure (Seca 201).

#### 2.3.4. Quality of Life, Sleep Quality, Daily Sleepiness Status and Fatigue

The interview method was used to complete all questionnaires. Quality of life was assessed by the Short-Form 36 Health survey (SF-36) [47]. The SF-36 is a 36-item survey that produces scores on eight constructs which are summarized in two components: the physical health and the mental health component. The total score is also calculated. Subjective sleep quality was assessed using the Pittsburg sleep quality index [48]. This is a 19-item questionnaire evaluating sleep quality and disturbances over the past month. A global score ranging from 0 to 21 is obtained, with a score > 5 suggesting poor sleep quality. The participants’ general level of daytime sleepiness was assessed with the Epworth Sleepiness scale [49]. This eight-item questionnaire consists of 8 self-rated items that measure a subject’s habitual likelihood of falling asleep during common activities of daily life. The score ranges from 0 to 24, while values >10 are considered to indicate significant sleepiness. Fatigue levels were evaluated by using the fatigue severity scale, which assesses the physical aspects of fatigue and their impact on the subject’s daily function. The score ranges 1–7, with lower scores indicating less fatigue [50]. Medical comorbidity was assessed using the Charlson Comorbidity Index, with higher scores indicating greater comorbidity [51].

### 2.4. Sample Size & Statistical Analysis

The planned sample size was based on the results of preliminary study (Neuroaspis PLP10^®^, proof-of-concept clinical trial) [35]. Twenty-three subjects were able to be randomized in each group including an extra 20% sample, in case any patient decided to drop out of—or was lost for—the follow-up. Eventually, eighteen of them in each group completed the study.

Statistical analysis was carried out with SPSS version 22 throughout the study on all those being analyzed. Welch’s t test was used to compare subject characteristics between the omega and placebo groups at baseline. A linear mixed model with a random effect for participant was used to evaluate differences in the dependent variables between groups throughout time, whilst it was adjusted for the age and education level of the participants. When significant, supplementation x time interactions were detected, post hoc analyses were used to locate differences using Bonferroni-adjusted pairwise comparisons. For within-subject pairwise comparisons, the magnitude of effect sizes was determined by calculating Cohen’s *d* (small effect = 0.20–0.49, medium effect = 0.50–0.79 and large effect ≥ 0.80). A *p* ≤ 0.05 was considered statistically significant. Data are presented as means ± SDs.

## 3. Results

The baseline main characteristics of the participants showed no significant differences between the two groups (Table 1). In general, the study supplementations were well-tolerated, and no serious adverse events or complications were reported from the participants. The most-commonly reported side effects from the participants included a fishy aftertaste (8 omega participants) and gastrointestinal symptoms—mostly diarrhea without pain for the first month (4 omega and 2 placebo participants).

### 3.1. Cognitive Function

**Figure 2:**
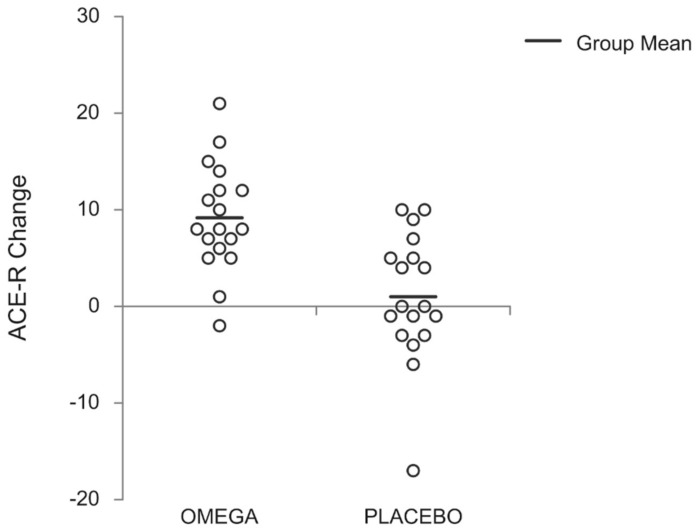
Individual Addenbrooke’s Cognitive Examination -Revised (ACE-R) change (pre-post) for each group.

Table 2 presents the results of the ACE-R and MMSE cognition tests. A significant supplementation effect (*p* = 0.028), a time effect (*p* < 0.001) and a supplementation x time interaction (*p* < 0.001) was found for the ACE-R score. Post-hoc tests revealed that the ACE-R score was significantly increased in mid- and post-supplementation compared to pre-supplementation in the omega group (*p* = 0.013, *d* = 0.58 and *p* < 0.001, *d* = 1.64; respectively), and it was greater than in the placebo group at these time points (*p* = 0.031 and *p* = 0.003, respectively). The individual ACE-R changes (pre to post) for each group are presented in Figure 2. Concerning the cognitive domains of ACE-R, there was a main effect of time for memory (*p* = 0.001) with the omega group eliciting a greater score at post-supplementation compared to their pre-supplementation score (*p* < 0.001, *d* = 1.06). A trend for significant supplementation effect (*p* = 0.067) and supplementation x time interaction (*p* = 0.086) was also observed for memory. The main effects for supplementation (*p* = 0.030) and time (*p* = 0.021) were observed for fluency, with a trend for significant supplementation x time interactions (*p* = 0.055), such that at post-supplementation the omega group had a greater score than the pre-supplementation value (*p* = 0.003, *d* = 0.82), and the corresponding placebo score (*p* = 0.004). Similarly, the main effects for supplementation (*p* = 0.047) and time (*p* = 0.001) were found for language without a significant supplementation x time interaction (*p* = 0.099), with the omega group having a greater score at mid- and post-supplementation compared to pre-supplementation (*p* = 0.039, *d* = 0.59 and *p* < 0.001, *d* = 0.91; respectively). Moreover, there was a significant difference between the two groups at post supplementation (*p* = 0.012). A significant interaction was found for visuospatial (*p* = 0.004), such that the omega group elicited greater score at post- compared to pre- and mid-supplementation (*p* = 0.017, *d* = 0.59 and *p* = 0.043, *d* = 0.50; respectively). In addition, there was a significant difference between the two groups at post supplementation (*p* = 0.009). There was a significant interaction for MMSE score (*p* = 0.011), with significant increase for the omega group post supplementation compared to the baseline value (*p* = 0.006, *d* = 0.71) and to the corresponding placebo value (*p* = 0.032).

The scores of the other cognitive function tests (TMT, STROOP and symbol cancellation) that were evaluated only at baseline and following 6 months of supplementation, are presented in Table 3. A significant time effect was found for STROOP word (*p* = 0.038), with the omega group having a greater score at post- compared to pre-supplementation (*p* = 0.029, *d* = 0.85). A significant time effect (*p* = 0.048) and supplementation x time interaction (*p* = 0.037) were found for the STROOP color score, such that the omega group elicited greater score at post- compared to pre-supplementation (*p* = 0.004, *d* = 0.83). A trend for significant interaction was found in STROOP color-word performance (*p* = 0.097), with the omega group showing a non-significant increase (*p* = 0.076, *d* = 0.45). Additionally, the omega group experienced a non-significant reduction in time completion of TMT B of medium magnitude (*p* = 0.103, *d* = 0.57). Main effects for supplementation (*p* = 0.003) and time (*p* = 0.001) were observed for the symbol cancellation test, with the omega group making less errors at post- compared to pre-supplementation (*p* = 0.001).

### 3.2. Functional Capacity

**Figure 3:**
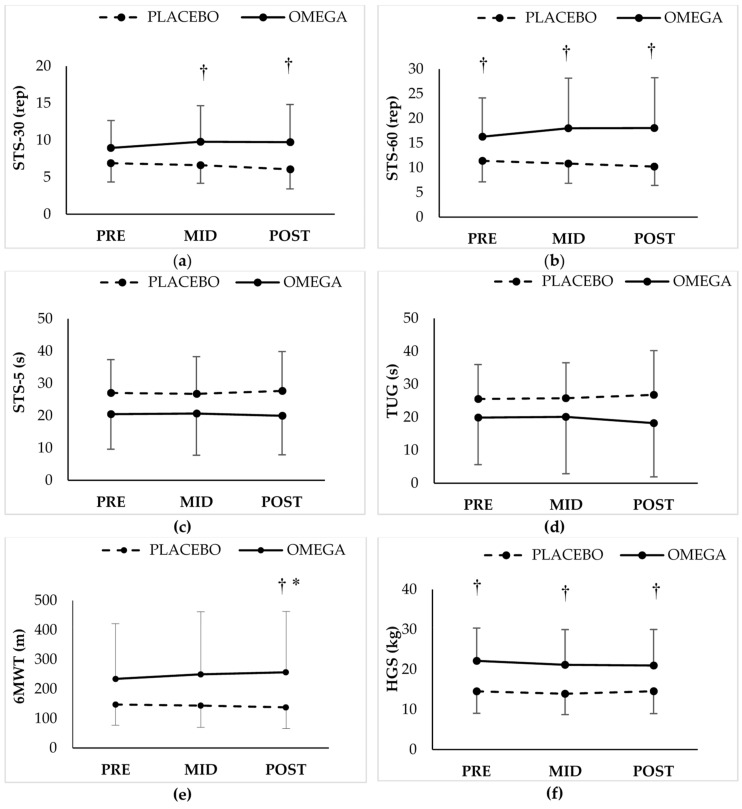
Functional tests ((**a**) Sit-to-stand-30; (**b**) Sit-to-stand-60; (**c**) Sit-to-stand-5; (**d**) Timed-up-and-go; (**e**) 6-min walk test; (**f**) Handgrip strength) for each group at pre, mid (3mo) and post (6mo) of placebo or omega supplementation. †: *p* ≤ 0.05 from the corresponding value in placebo; *: *p* ≤ 0.05 from pre-supplementation in the same group. STS: Sit-to-stand; TUG: Timed Up and Go; 6MWT: 6-min walk test; HGS: Handgrip Strength.

All functional capacity tests are presented in Figure 3. There was a significant interaction between supplementation and time for STS-30 (*p* = 0.050) and a significant supplementation effect (*p* = 0.023), such that the omega group performed more sit-to-stand cycles at mid- and post-supplementation assessments compared with the placebo group (*p* = 0.020 and *p* = 0.006, respectively). Similarly, a significant interaction (*p* = 0.032) and supplementation effect (*p* = 0.007) was found for STS-60 with the omega group completing more cycles at mid- and post-supplementation assessment compared with the placebo group (*p* = 0.005 and *p* = 0.002; respectively). No significant changes were found for STS-5 and TUG. A significant supplementation x time interaction was observed for 6MWT (*p* = 0.028), with the omega group achieving longer distance at post-supplementation compared to the pre-supplementation value (*p* = 0.027, *d* = 0.45), and compared to the corresponding placebo group value (*p* = 0.051). A main effect of group was found in HGS (*p* = 0.003), with the omega group obtaining greater values at all time points (*p* < 0.05).

### 3.3. Body Composition

Body composition indices are presented in Table 4. A significant effect of supplementation was found in % total body fat (*p* = 0.027), with the omega group having lower values at mid- and post-supplementation time points compared to the placebo group (*p* = 0.016 and *p* = 0.030, respectively). A trend for significant interaction was observed for weight (*p* = 0.060), lean body mass (*p* = 0.069), lean mass index (*p* = 0.090) and waist circumference (*p* = 0.065).

### 3.4. Questionnaires

The scores of all questionnaires are presented in Table 5. A significant supplementation x time interaction was found for fatigue (*p* < 0.001), such that the omega group reported less fatigue levels at mid- and post-supplementation time points compared to pre levels (*p* = 0.004, *d* = 0.64 and *p* = 0.001, *d* = 0.67; respectively). There was a main effect of time for sleep quality (*p* = 0.033), with a lower score presented at mid- (*p* = 0.018, *d* = 0.65) and post- (*p* = 0.067, *d* = 0.48) time points compared to pre-supplementation in the omega group. A significant supplementation x time interaction was observed for sleepiness (*p* = 0.007), with the omega group showing a trend for a significant reduction from pre to post-supplementation (*p* = 0.077, *d* = 0.44), and the placebo group presenting a marginal increase for the same time period (*p* = 0.063, *d* = 0.51). Regarding quality of life, there was a significant interaction (*p* = 0.007) and a time effect (*p* = 0.027) for the physical health component, with the omega group eliciting greater values at mid- and post-supplementation time points compared to pre-supplementation levels (*p* = 0.001, *d* = 0.69 and *p* = 0.005, *d* = 0.65; respectively).

## 4. Discussion

The results of the present study showed that a high-dose of omega-3 and omega-6 fatty acids with antioxidant vitamins supplementation improves cognitive function and functional capacity in older adults with MCI. In addition, favorable improvements for the participants receiving this nutritional supplement were shown in fatigue, the physical health component of quality of life, and daily sleepiness.

MCI represents an intermediate state of cognitive function between the changes seen in aging and those fulfilling the criteria for dementia [52]. Persons with MCI are at an increased risk for developing dementia [53]. It has been suggested that participants with MCI are more benefited in cognition than those with Alzheimer’s disease with PUFAs supplementation [54]. The findings from this study suggest that supplementation with omega fatty acids under appropriate formulations and dosage in older adults with MCI might improve cognitive function. Bo et al. demonstrated that six months of supplementation with 480 mg DHA and 720 mg EPA per day could improve perceptual speed, space imagery efficiency and working memory in older adults with MCI, whereas Sinn et al. showed that 6 months’ supplementation with fish oils rich in DHA (1.55 g DHA and 0.40 g EPA per day) improved fluid thinking ability [55,56]. In the present study, the global cognition score of both cognition tests performed (ACE-R and MMSE) showed a greater improvement in the omega supplementation group compared with placebo. Therefore, the findings of the present study—with the specific fatty acid formulation along with specific antioxidant vitamins and the new high dose—confirm the findings of both studies mentioned above, and add further knowledge on more beneficial effects on cognitive function. In addition, the cognitive domains of memory, fluency, language and the visuospatial were all improved in the omega group compared to baseline values, which is of great interest since these cognitive domains are usually impaired in MCI [34]. Moreover, because executive function is significantly impaired in MCI, more tests were performed to examine the impact of omega supplementation on various aspects of executive functions such as inhibition and cognitive flexibility [57]. The results of the present study showed an improvement in these tests for the omega group, albeit, only the STROOP word, the STROOP color performance and the symbol cancellation test showed significant differences. However, taken together, the overall improvement of global cognition observed in the current study may have important clinical implications in terms of preventing or delaying the progression of cognitive decline associated with aging with potential applications to other neurodegenerative diseases as well.

The higher dose of DHA given in the present study compared to other studies, or the synergy of the combined ingredients as a cocktail formulation, might partly explain the observed positive effects on cognition. Quantitatively, DHA is the most important omega-3 PUFA in the brain, its potential neuroprotective properties could help to protect older adults who are at risk of developing cognitive decline and dementia [8,16]. Furthermore, and as previously noted, cell membrane fatty acid composition—especially of the brain—can be modified with dietary supplementation, but it is age dependent (much more is needed in elderly) [9]. It has also been proposed that DHA is important in regulating neurogenesis, increasing neural synapses and membrane fluidity and protecting against neuronal damage [58]. In addition, ageing is associated with a deficiency in antioxidants in the body and an aberrant redox homeostasis compared to in younger adults [59]. Inflammation is also a characteristic of many neurodegenerative diseases like dementia. Conclusively, we may also suggest that the anti-inflammatory and antioxidant properties of EPA and DHA—along with the LA and GLA in combination with the specific antioxidant vitamins found in the given cocktail supplement formula used—may have holistically and synergistically contributed to the cognitive-enhancing effect; potentially through the reduction in both oxidative stress and inflammation [6,16].

Brain health is strongly linked to physical health, and physical function is, to a large extent, cognitively mediated [60]. Evidence has demonstrated that the impaired functional capacity that leads to physical frailty is associated with worse cognitive trajectory among participants with MCI [61]. However, limited data are present in the literature regarding the effect of omega fatty acids or any other natural formula supplementation on functional capacity in the elderly and especially with regards to participants with MCI. To our knowledge, this is also the first study to evaluate and demonstrate an increase in the distance walked on a 6MWT following an omega fatty acid and/or a PUFA-based formula supplementation in older adults. The 6MWT has been proposed as a valuable tool to assess physical endurance in the elderly [62]. The 9.6% improvement found in the present study is of great importance, since performance on the 6MWT is independently associated with all-cause mortality in older adults [63]. The consumption of the specific formula significantly increased the completed sit-to-stand cycles in the STS-30 and STS-60 tests as well, and the improvement was observed from the first three months of supplementation. These tests measuring lower body power, balance and endurance are the most important physical performance clinical tests, because they relate to the most demanding daily life activities [23]. In contrast, the study of Logan and Spriet did not find any improvement in STS-30 following 3 g/d of EPA and DHA intake for 12 wk in older females [30]. Interestingly, Rodacki et al. showed that fish oil supplementation (2 g/d for 90 days) during a strength-training study on elderly women induced further improvements in the chair-rising test, suggesting that the use of this high concentration PUFA-based supplementation potentiates the neuromuscular system [64].

However, we should note that the reduction observed in TUG (1.7 s, 8%) was not statistically significant, in contrast with a previous study that found a smaller but significant reduction in TUG time (0.5 s, 7%) [30]. Moreover, the same study along with others did not find any effect of supplementation on handgrip strength, similar to the present study [28,29]. In contrast, Smith et al. reported that 6 months supplementation with 1.86 g/d EPA and 1.50 g/d DHA in healthy older adults led to significant beneficial effects on handgrip strength, and upper- and lower-body 1-RM muscle strength [27]. A possible explanation for this confounding resulting outcome could be related to the muscle group that was evaluated. If there is a minimum stimulus to elicit strength increases, the large degree of inactivity of the participants in the present study, most of whom resided in care homes, could explain the lack of strength improvements in the upper body [30].

Unfortunately, no direct evaluation of muscle mass was performed. However, a trend for significant increase in the estimated lean body mass was found, indicating a possible increase in muscle mass. In support of this notion, Smith et al. found a 3.5% difference in muscle volume between omega–3 PUFA vs control groups after 6 months supplementation. Another study showed that 12 weeks of fish oil supplementation increased lean mass in healthy community dwelling older females [27,30]. Given the age-related loss of muscle strength, quantity or quality and the lower physical performance (a condition called sarcopenia) that could impair the ability to perform activities of daily living, the use of omega fatty acids might be an alternative therapeutic agent [65,66]. Counting all aforementioned data, along with the observed improved functional capacity and the absence of serious adverse events, we can potentially presume that this specific nutrition formula supplementation can hugely benefit the geriatric population.

Regarding the procedures underlying these improvements, the exact molecular mechanisms that mediate improved skeletal muscle mass and functionality with omega fatty acid intake and/or in combination with specific vitamins (as in the present formula used) have not been fully elucidated. The most-commonly proposed mechanism to underpin the anabolic action of PUFA is via the incorporation of EPA/DHA and LA/GLA into the membrane phospholipids of the sarcolemma and intracellular organelles as well as at the cell membranes at the inflammation sides [35,67,68]. Furthermore, the presence of the specific antioxidant vitamins can modulate the overall positive effect by limiting the presence of the free radicals (both reactive oxygen species (ROS) and reactive Nitrogen species (RNS) as a result of the ongoing oxidative stress which can potentially affect muscle function and age-related sarcopenia [35,69]. This structural change of the muscle membrane may activate intracellular signaling proteins (e.g., mTORC1-p70S6k1) that upregulate muscle protein synthesis, thus modulating muscle mass [26,67,68]. The positive effects on muscle function could also be related to the proposed beneficial effects of PUFAs on mitochondrial and neuromuscular function. Previous animal studies have found that fish oil supplementation led to an increased contractility due to enhanced sensitivity to acetylcholine [70]. Furthermore, n3-PUFA supplementation in older adults resulted in reduced mitochondrial oxidant emission rates and increased the expression of genes involved in muscle mitochondrial function [25,71]. On the other hand, supplementation with omega fatty acids could lead to physiological and metabolic adaptations which could favor functional capacity like improved endothelial function and increased metabolic rate and fat oxidation [30,72]. Even though there is contradictory evidence regarding the effects of antioxidant intakes on exercise performance, it has been suggested that individuals with antioxidant deficiency or with high oxidative stress levels may benefit from the antioxidant treatments [73,74,75]. In support of this assertion, a recent study has shown that antioxidant supplementation decreased oxidative stress and improved physical performance in old but not in young adults [59]. Therefore, we may possibly conclude that the seniors enrolled in the present study might have been benefited from the specific antioxidant vitamins found in the formula used in a way that has partially contributed to the observed augmentation on functional capacity. However, more studies are needed to investigate this assumption. On the other hand, it is well known from the literature that free radicals (both ROS and RNS) are, generally, strong contributors to the aging evolution. For this reason, we have used the aforementioned formula including high concentration of specific antioxidant vitamins as well.

An interesting finding of the present study was that the improvement in functional abilities observed in the participants of the omega group was further reflected in quality of life, where the physical health component was increased. Reduced daily functioning has been associated with reduced quality of life in adults with MCI, therefore targeting functional symptoms may be an effective way to improve quality of life for these individuals [76]. In addition, the finding of reduced fatigue from the first 3 months of omega supplementation is of great importance, since fatigue is a strong predictor of functional limitations, disability, mortality and other adverse outcomes in aging populations [77]. Furthermore, both groups had low levels of sleep quality before the intervention, while sleepiness and sleep quality showed improvement (albeit not statistically significant) following the specific supplementation. Poor sleep duration and quality have been associated with impaired cognitive function, declining functional status and quality of life, as well as greater rates of depression [78,79]. Thus, even a minor improvement may have clinical significance on this population. To date, to our knowledge, only a few clinical trials have been performed evaluating the benefits of PUFAs on quality of life, sleep or fatigue for the elderly. A previous 18-month clinical study using 1720 mg DHA and 600 mg EPA per day did not find any improvement in quality of life or sleep in cognitively healthy community-dwelling adults [80]. Another study reported that increased DHA in erythrocyte membrane (that means increased consumption of DHA rich diet) was significantly associated with improved self-reported physical functioning on the SF-36 in older adults with MCI [56].

One of the limitations of the present study was the uncontrolled dietary intake and physical activity levels of the participants during the study. More than half participants of each group were long-term care facility residents; they were physically inactive and did not participate in any organized physical activity, whilst they had a standard weekly nutritional menu. However, we can suggest a homogeneity of the daily activities and the daily diet opportunities of the enrolled studied population. This can possibly be considered as a positive parameter strengthening the overall study outcome. Moreover, the high concentration of the PUFAs in the daily dose of the formula used was able to calibrate any daily nutritional habit of the enrolled population [35]. Other limitations of the study were the lack of a direct evaluation of the participants’ muscle mass, and the low number of participants completing the TMT, STROOP and symbol cancellation tests. Moreover, the small sample size in each group should also be considered as a limitation in our study. On the other hand, the strengths of the present study—including the comprehensive assessment of cognitive and physical function in addition to various questionnaires regarding the well-being of cognitive impaired older adults, the robust clinical protocol followed with specific inclusion criteria and according to the international guidelines, and the population homogeneity—give originality and additional strength to the quality of the overall outcome. It should be further noted that this is the first study using such a high dose of specific PUFAs along with specific antioxidant vitamins in elderly adults for a period of six months. The fact that no serious side effects were reported is additionally encouraging for the use of this specific supplement. However, future clinical studies with larger samples sizes and longer study durations are needed to affirm our results. Whether the efficacy of such formulations on skeletal muscle is influenced by sex remains yet to be investigated.

## 5. Conclusions

The present study demonstrated that a high-dose of specific omega-3 and omega-6 fatty acids supplementation, in combination with specific antioxidant vitamins, can be a potential nutritional modality for the prevention or possibly treatment of cognitive impairment and functional decline; thereby improving independence and quality of life for older individuals with MCI.

## Figures and Tables

**Table 1:** Baseline characteristics of participants.

	PLACEBO	OMEGA
N	18	18
Sex (F/M)	12F/6M	10F/8M
Age (years)	81.2 ± 5.3	77.4 ± 9.2
Long-term care facility residents (*n*)	13	12
ACE-R	63.9 ± 10.2	69.4 ± 11.2
Education (years)	9.3 ± 4.1	8.4 ± 4.1
Charlson comorbidity index	2.4 ± 1.2	1.8 ± 1.7

N: number of participants; F: females; M: males; ACE-R: Addenbrooke’s Cognitive Examination -Revised test.

**Table 2:** ACE-R and MMSE tests at pre, mid (3mo) and post (6mo) of placebo or omega supplementation.

	PLACEBO			OMEGA		
	PRE	MID	POST	PRE	MID	POST
ACE-R (total score)	63.9 ± 10.2	63.8 ± 9.9	64.9 ± 13.3	69.4 ± 11.2	73.7 ± 15.7 †*	78.6 ± 14.0 †*#
Attention/orientation	14.5 ± 2.8	14.4 ± 3	14.6 ± 3.7	15.1 ± 2.7	15.4 ± 2.8	16.4 ± 2.4
Memory	14.1 ± 4.9	13.4 ± 4.6	15.0 ± 5.6	15.8 ± 3.7	17.3 ± 5.9	19.1 ± 5.1 *
Fluency	3.6 ± 2.5	4.2 ± 2.4	3.7 ± 2.2	5.2 ± 2.3	6.0 ± 3.0	6.6 ± 3.0 †*
Language	19.2 ± 3.5	19.8 ± 3.6	19.7 ± 3.1	20.3 ± 4.0	21.7 ± 4.5 *	22.5 ± 3.7 †*
Visuospatial	12.6 ± 2.6	12.1 ± 2.5	11.9 ± 2.7	13.1 ± 2.5	13.2 ± 2.3	14.1 ± 2.1 †*#
MMSE	23.6 ± 3.2	23.3 ± 3.5	22.9 ± 4.6	24.3 ± 3.5	25.2 ± 3.9	26.1 ± 3 †*

†: *p* ≤ 0.05 from the corresponding value in placebo; *: *p* ≤ 0.05 from pre-supplementation in the same group; #: *p* ≤ 0.05 from mid-supplementation in the same group. ACE-R: Addenbrooke’s Cognitive Examination -Revised; MSSE: Mini-Mental State Examination.

**Table 3:** Trail Making Tests (TMT), Stroop Color and Word (STROOP) tests, and cancellation tests at pre and post (6mo) of placebo or omega supplementation.

	PLACEBO			OMEGA		
	*n*	PRE	POST	*n*	PRE	POST
TMT A (s)	14	251.5 ± 148.7	236.4 ± 146.7	17	168.5 ± 142.6	155.7 ± 157
TMT B (s)	8	420.5 ± 203.3	415 ± 162.8	10	263.3 ± 167.4	201.6 ± 142.2
STROOP word (score)	12	48.6 ± 15.8	51 ± 15.2	14	55.2 ± 23.3	61.3 ± 24.7 *
STROOP color (score)	12	33.3 ± 12	33.1 ± 13.8	14	31.1 ± 16.9	36.9 ± 17.8 *
STROOP color-word (score)	12	14.3 ± 6.9	12.5 ± 9.1	14	14.4 ± 9.8	19.1 ± 13.3
STROOP interference (score)	12	−4.9 ± 5.9	−6.9 ± 8.4	14	−5.2 ± 8.6	−3.6 ± 11.1
Symbol cancellation (errors)	13	53.4 ± 6.7	50.0 ± 7.6	14	37.6 ± 15.8†	31.6 ± 14.5 *†

*n* indicates the total participants that were included in the analysis for each test; *: *p* ≤ 0.05 from pre-supplementation in the same group; †: *p* ≤ 0.05 from the corresponding value in placebo.

**Table 4:** Body composition indices at pre, mid (3mo) and post (6mo) of placebo or omega supplementation.

	PLACEBO			OMEGA		
	PRE	MID	POST	PRE	MID	POST
Body mass (kg)	70.8 ± 19.2	70.2 ± 19.4	70.0 ± 19.3	71.5 ± 13.0	72.1 ± 14	72.1 ± 13.7
Body mass index (kg/m^2^)	33.9 ± 7.7	33.6 ± 7.8	33.5 ± 7.8	33.5 ± 6.3	33.8 ± 6.6	33.8 ± 6.6
Trunk fat (%)	38.5 ± 10.3	37.8 ± 9.8	38.0 ± 10.3	35.4 ± 9.7	34.5 ± 9.7	35.4 ± 9.5
Total body fat (%)	41.1 ± 8.3	41.3 ± 7.8	40.5 ± 7.5	37.1 ± 6.2	36.0 ± 6.8 †	35.9 ± 7.3 †
Total body water (%)	50.8 ± 5.8	50.9 ± 5.8	51.5 ± 5.2	53.1 ± 6.0	53.8 ± 6.9	54.1 ± 7.0
Lean body mass (kg)	42.8 ± 12.3	42.2 ± 12.3	42.6 ± 12.1	44.7 ± 10.0	45.9 ± 11.5	45.9 ± 11.2
Fat mass index (kg/m^2^)	11.9 ± 4.2	11.8 ± 4.2	11.6 ± 4.1	10.2 ± 2.6	9.9 ±2.7	9.9 ± 2.8
Lean mass index (kg/m^2^)	16.7 ±3.7	16.5 ± 3.7	16.7 ± 3.7	17.1 ± 3.1	17.5 ± 3.6	17.5 ± 3.4
Waist circumference (cm)	107.9 ± 12.8	107.2 ± 12.6	107.0 ± 13.2	101.2 ± 10.5	103.0 ± 10.6	100.1 ± 11.7
Hip circumference (cm)	108.4 ± 13.3	107.6 ± 13.5	107.6 ± 13.8	105.1 ± 9.9	103.7 ± 10.0	103.6 ± 10.4

†: *p* ≤ 0.05 from the corresponding value in placebo.

**Table 5:** Fatigue, sleep quality, sleepiness and quality of life at pre, mid (3mo) and post (6mo) of placebo or omega supplementation.

	PLACEBO			OMEGA		
	PRE	MID	POST	PRE	MID	POST
Fatigue	4.2 ± 1.5	4.4 ± 1.5	4.6 ± 1.7	4.5 ± 1.8	3.8 ± 1.7 *	3.7 ± 1.9 *
Sleep quality	8.5 ± 3.5	8.0 ± 3.2	8.5 ± 3.4	8.7 ± 5.1	7.1 ± 4.9 *	7.4 ± 4.7
Sleepiness	6.9 ± 4.2	7.3 ± 3.5	8.5 ± 4.0	7.6 ± 5.1	6.6 ± 3.5	6.1 ± 3.0
Quality of life						
Physical health component	47.5 ± 19.9	47.2 ± 17.7	45.7 ± 16.8	50.8 ± 22.0	60.8 ± 21.3 *	59.2 ± 23.2 *
Mental health component	58.7 ± 18.6	60.9 ± 21.2	61.2 ± 21.2	57.4 ± 20.3	60.7 ± 21.1	61.9 ± 20.5
SF-36 total score	51.5 ± 19.4	52.6 ± 19.8	52.3 ± 18.9	53.1 ± 20.3	60.4 ± 21	60.3 ± 22.1

*: *p* ≤ 0.05 from pre-supplementation in the same group.
